# Translation and cultural adaptation of the Illness Invalidation Inventory for use in Sweden

**DOI:** 10.1186/s40359-014-0057-9

**Published:** 2014-12-24

**Authors:** Pirjo Järemo, Maria Arman

**Affiliations:** Department of Neurobiology Care Sciences and Society (NVS) Karolinska Institutet, Huddinge, S-141 83 Sweden

**Keywords:** Invalidation, Questionnaire, Translation, Cultural adaptation

## Abstract

**Background:**

This study describes the translation and cultural adaptation procedure and guidelines for the Illness Invalidation Inventory for use in Sweden. Invalidation includes responses to negative social interactions and to the lack of positive social interactions, responses that can negatively affect health and suffering. Although invalidation is a recognized phenomenon, in Sweden no instruments exist that describe and measure invalidation. To this end, this study evaluates the translation and adaptation of the Illness Invalidation Inventory as an instrument for measuring invalidation in a Swedish context.

**Methods:**

Internationally recognized ten-step guidelines were used. Both forward and back translations were performed. Patients from a patient organization for chronic pain were recruited and cognitive interviews were performed using concurrent think aloud protocols, probing techniques and observations of behaviour. Analysis of data collected from cognitive interviews was inspired by the generic response model and a centralized review procedure and thorough documentation was emphasized.

**Results:**

Although difficulties regarding concepts were found, these issues were solved during the process. The Swedish version contains the same number of items as the original questionnaire. Four of eight items required revision after cognitive interviews.

**Conclusions:**

The study highlights the importance of using guidelines to produce translations and to ensure validity and results. The results indicate that the Illness Invalidation Inventory can be used in Sweden to measure invalidation.

## Background

Living with pain is an intensely challenging and overwhelming condition that intrudes in unpredictable ways on most aspects of life (McBeth et al. 2010; Choy et al. 2010). As some pain does not exhibit external clinical signs, this situation can be described as an invisible illness (Rodham et al. 2010). Patients with invisible and medically unexplained conditions often have problems with credibility thereby affecting their access to and experience with health care (Lempp et al. 2009). The decline of patients’ perceived quality of life is described as dramatic with loss of social support and lack of health care support (Schoofs et al. 2004). Previous studies also show that such offensive and degrading treatment, especially in the context of health care, leads to increased patient suffering (Arman et al. 2004; Jaremo and Arman 2011).

In an earlier study exploring patients’ beliefs about their illness, we found that patients had experienced offensive and degrading treatment both in their private life and in their contact with authorities (Jaremo and Arman 2011). Wanting to measure these experiences, we found only one questionnaire that measures invalidation emanating from different sources – the Illness Invalidation Inventory (Kool et al. 2010). Kool et al. (2009) identified the definition and structure of invalidation perceived by patients with rheumatic diseases as experiences of active negative social responses and as lack of positive social responses.

There is no consensus regarding terms and methods of translation and cultural adaptation of questionnaires (Acquadro et al. 2008; Maneesriwongul and Dixon 2004; Wild et al. 2005). However, translating a questionnaire is not enough: a questionnaire should be adapted so it is in a relevant and comprehensive form. The questionnaire should work irrespective of culture by maintaining intent and meaning of the items (Flaherty et al. 1988). Furthermore, the validity of studies using translated questionnaires could be compromised if there is a lack of or insufficient description of the procedures (Maneesriwongul and Dixon 2004).

Several guidelines, however, recommend multiple techniques to be used in all cross-cultural research (Acquadro et al. 2008; Maneesriwongul and Dixon 2004). Existing guidelines agree that cognitive debriefing is needed to ensure that the intended patient group can adequately comprehend the translation.

Patients’ perspectives of their illness have been investigated from an holistic approach, including the impact of their illness beyond the medical view on their body (Todres et al. 2007). Instruments measuring patients’ experiences from interactions with persons around them during their illness would allow health care professionals to acknowledge and better understand the aspect of invalidation. This aspect could also be included in programs for pain-related rehabilitation and in health care measures for patients.

### Aim of the study

This study describes the procedure for translation and cultural adaptation of the Illness Invalidation Inventory for use in Sweden.

## Methods

“The Illness Invalidation Inventory (3*I)” questionnaire, developed to measure invalidation related to illness, was translated from English into Swedish (Kool et al. 2010). The questionnaire assesses the extent to which people experience invalidation (five items related to discounting and three items related to lack of understanding) from five sources: spouse, family, work, medical professionals, and social services. Respondents indicate on a five-point Likert-type scale (1 = never to 5 = very often) how often during the past year they experienced invalidation. The questionnaire was constructed in populations with rheumatic diseases and has been found to be a reliable, valid, and brief instrument for assessing patients’ perceptions of invalidation.

Since multiple methods were recommended to yield best results (Acquadro et al. 2008; Maneesriwongul and Dixon 2004), we chose to use the ISPOR (Wild et al. 2005) guidelines (Table 1), which meets these criteria. Ten translators were recruited to perform the translations. For cultural adaptation, 11 respondents were recruited for cognitive interviews from a patient organization for chronic pain and they represent demographic variety with respect to gender, age, education, and work status (Table 2). After the staff of the patient organization asked who wished to be included, the researcher called the patients, asked them if they wanted to participate, and provided oral and written informed consent to participate in the study. These respondents were chosen because they were similar to the intended target population of a future study where the translated questionnaire will be used. Cognitive interviewing allows understanding the questionnaire from the respondent’s perspective rather than the researcher’s and is most valuable when presenting questions that are sensitive and intrusive (Drennan 2003), as some questions in the Illness Invalidation Inventory might be perceived as such. Cognitive interviewing can help researchers adapt a questionnaire developed for use in one culture to another and is advocated by several guidelines (Acquadro et al. 2008; Collins 2003; Beatty and Willis 2007; Knafl et al. 2007). The interviews were analysed and response problems were categorised as problems either in the process of comprehending (understanding the words and what is being requested, how to provide this data), performing the task (retrieval and evaluation of data) or formatting the response (how to map the recalled data with response options) (Conrad and Blair 1996). Since the most centralized and rigorous procedures provide best outcomes (Acquadro et al. 2008; Wild et al. 2005), the importance of keeping a centralized review procedure and thorough documentation of the procedure was emphasized.

**Table 1:** **Guidelines for translation and cultural adaptation process for patient-reported outcomes measures according to Wild et al.** (2005)

Translation procedure	
1. Preparation	
2. Forward translation	
3. Reconciliation	
4. Back translation	
5. Back translation review	
Cultural adaptation procedure	
6. Harmonization	
7. Cognitive debriefing	
8. Review of cognitive debriefing results and finalization	
9. Proof reading	
10. Final report

**Table 2:** **Characteristics of patients with chronic pain (n = 11)**

Gender: female, *n*	9
Male, *n*	2
Age (years), mean, (range)	51.5 (38–65)
Education level, *n*	
Compulsory school	1
Upper secondary school	8
Academic degree	2
Years since diagnosis, mean	12.1
Work status, *n*	
Working full time	0
Working part time	6
On sick leave	1
Retired	2
Sickness pension	1
Early retirement	1
Native language, *n*	
Swedish	10
Finnish	1
Diagnosis, *n*	
Fibromyalgia	10
Cervicobrachial syndrome	1

### The cross-cultural adaptation process

**Figure 1:**
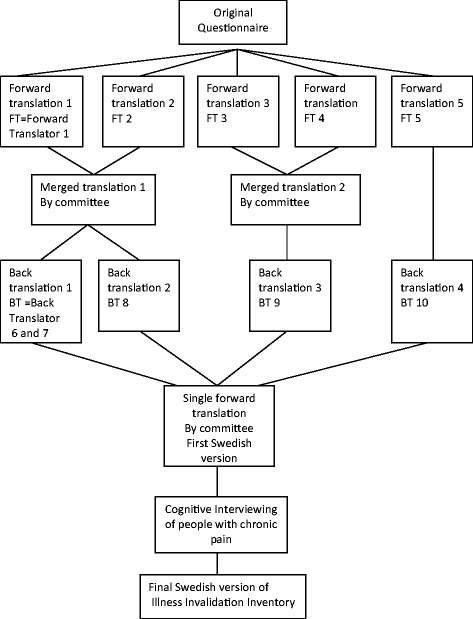
**Overview of the translation process.**

The cross-cultural adaptation process consists of two parts: the translation and the cultural adaptation (Figure 1).

### Translation procedure

Step 1: *Preparation.* Permission was obtained from the instrument designer (Marianne B. Kool) to translate the English version of the questionnaire (called the original in this study) into Swedish and to consult the designer during the process (e.g., intentions of items and instructions). Eleven respondents were contacted for the cognitive interviews and ten bilingual translators were selected to translate the instrument into their native language. The goal was to achieve conceptual equivalence between the original and the Swedish version to ensure the subjective items in the original were properly constructed and to obtain a translated version easily understood by the general population. Due to the sensitive and intrusive concepts in the items several translations were preferred in order to reduce translation bias. A review committee was formed consisting of a PhD student, an associate professor, a bilingual physician and a teacher in French and Swedish. Committee decisions were made by consensus with final responsibility on the two first members (the authors).
Step 2: *Forward translation*. The two first forward translations were made by the PhD student (Forward Translator 1 = FT 1) and the associate professor in the caring sciences (FT 2). Three additional forward translations were conducted by a senior credit analyst (FT 3), an assistant professor in English (FT 4) and a bilingual physician (FT 5).
Step 3: *Reconciliation*. To avoid translation bias due to personal style four of the forward translations were merged into two and the fifth one was not merged in order to obtain several alternatives instead of one forward translation. The committee reviewed the translated items by comparing them with the original questionnaire. Decisions were made by consensus; the most frequent translation was chosen while considering the conceptual equivalence.
Step 4: *Back translation*. The first merged forward translation was back translated twice. The first back translation was conducted collaboratively by two professional translators – natives from Australia and USA working as professional translators with experience translating in the health care field (Back translator 6 = BT 6 and Back translator 7 = BT 7). The second back translation was made by a (native British) teacher of English (BT 8). The second merged forward translation was back translated only once by a British editor (BT 9). The fifth forward translation, which was not merged, was back translated by a scientific expert from the Council of Europe committees (native British) (BT 10).
Step 5: *Back translation review*. The back translations were compared with the original by the committee and a preliminary Swedish version of the questionnaire was produced. Items or instructions with problematic wording or conceptual ambiguities were identified, preliminary translation solutions were found, and the designer was consulted on issues regarding design and conceptuality.

### Cultural adaptation procedure

Step 6: *Harmonization*. According to the instrument designer, the questionnaire was translated into English from a Dutch version and the English version was used to translate uniformly into French, German, Spanish and Portuguese by the designer and a small group of experts. All translated versions were received from the designer after the cognitive interviews and the committee (complemented with a teacher of Spanish) made simple comparisons between the Swedish version and the other versions (except the Portuguese) to evaluate whether the Swedish items corresponded with those in the other versions. The committee members indicated their evaluation by writing “Yes” or “No” after each item followed by comments.
Step 7: *Cognitive debriefing*. Cognitive interviews were performed using concurrent think aloud, probing technique and observation of behaviour as described by Willis (Willis 2005) to elicit information about potential problems in the translated version of the questionnaire. The face-to-face interviews were conducted at a location chosen by the respondents (interviewers or respondent’s workplaces or respondent’s home). All interviews were tape-recorded with the respondent’s permission. An interview guide with the following probes was used: paraphrasing, defining meanings of words used in items, and explaining responses in order to find problems of understanding the question, performing the task and response formatting. In addition, general probes were used to identify difficulties in instructions, the relevancy of the content, and an overall impression of the questionnaire. The respondents’ behaviour was observed and perceived difficulties experienced with the questionnaire were directly questioned. Using an iterative approach (Willis 2005), the interviewer conducted three rounds of interviews until the responses were deemed redundant. The number of respondents in the first three rounds were four and in the last round three. After each round, alterations were agreed on and the committee provided a new translation that was tested in the next round. The tape-recorded interviews were revisited by one committee member.
Step 8: *Review of cognitive debriefing results and finalization.* The committee agreed on modifications based on the results from the cognitive interviews. Reviewing responses confirmed findings of three problem types similar to Conrad’s (Conrad and Blair 1996) description: 1) comprehension of the question; 2) recall of information; and 3) response formatting.
Step 9: *Proof reading.* The committee proofread the finalized translation.
Step 10: *Final report.* Description of the methodology used included translator and respondent characteristics, translations, item and section comments and decisions undertaken.

### Ethical considerations

Respondents participated after informed consent. This translation process is part of a research project approved by the regional Research Ethics Committee of Stockholm aimed at describing and understanding illness beliefs in patients with chronic widespread pain.

## Results

This section reports findings from the steps of the translation and the cross-cultural adaptation process (Table 3).

**Table 3:** **Cognitive interview respondents’ (n = 11) commenting on the items and changes of items after pre-test**

**Items in original wording**	**Commented on the item (n)**	**Difficulties due to understanding (n)**	**Difficulties due to task performance (n)**	**Difficulties due to response formatting (n)**	**Changed in the Swedish version after pre-test**
1. … finds it odd that I can do much more on some days than on other days	6		4	2	Yes
2. …thinks I should be tougher	6		4	2	Yes
3. … takes me seriously	3		2	1	No
4. … gives me unhelpful advice	8	2	5	6	No
5. …understands the consequences of my health problems or illness	8		5	3	No
6. … makes me feel like I am an exaggerator	3	3		3	Yes
7. … thinks I can work more than I do	8		7	1	No
8. … gives me the chance to talk about what is on my mind	6	5		1	Yes

### Forward translation, back translation, and first committee review

Consensus was reached about the wording in the forward translations of items. The most frequent translations were chosen and conceptual equivalence was considered. The back translation of items was considered to correspond well. The changes done in this phase are listed below.

- Item 4: In “…gives me unhelpful advice”, “unhelpful” was translated in various ways by the Swedish translators (e.g., “unnecessary”, “unwelcome”, and “worthless”). Due to lack of equivalent expression and after contact with the designer about the meaning, the construction of the sentence was altered to “gives me advice that is of no help”.
- Item 6: In “…makes me feel like I am an exaggerator”, the expression “an exaggerator” was replaced with “makes me feel like I am exaggerating” since there is no direct translation.
- Item 8: There were several different translation options for “gives me the chance to talk about what is on my mind”. Since there is no corresponding phrase in Swedish, “talk about what I am thinking” was selected and talk was initially translated to “*tala*” (Swedish).
- In instructions for section 1 (spouse or partner), “partner” has a broader interpretation in English than in Swedish and was replaced with “permanent companion”.
- In instructions for section 2 (family), “family” only refers to the closest members in the core family, so it was changed to “family and relatives” to reflect the target language’s meaning. (In other cultures “family” can be interpreted in a broader sense.) With approval of the designer, the description of the source was shortened to “children, parents, and other relatives”.
- In instructions for section 5 (social services), “social services” was substituted with the authorities that organize the corresponding functions in Sweden.

### Cultural adaptation process and second committee review

A second committee review during and after the cognitive interviews resulted in contacts with the instrument designer about concepts and their intentions, which were questioned during the interview rounds and finally changed (Table 3). Items and instructions that were revised are listed below:

- Item 1: Most translators had used the Swedish word for “strange” in the translation of “odd” in “…finds it odd that I can do much more on some days than on other days”, which the committee felt did not fit the context, so “*underligt*” (Swedish) was chosen.
- Item 2: “Tough” was translated as “hard/strong” in “…thinks I should be tougher” the committee decided after contact with designer to use “endurable” for conceptual equivalence. “Endurable” was understood as “patient” and was changed to “endure more”.
- Item 3: The item “…takes me seriously” was well understood but presented some hesitation in choosing representative persons and situations to illustrate this reaction.
- Item 4: “…gives me unhelpful advice” presented some hesitation. That is, because of the negation it had to be read several times and was finally understood in the right way and interpreted in a positive way, which means that it was better to get advice even though the advice was unhelpful as providing advice irrespective of its usefulness at least demonstrated a caring attitude. Response formatting presented difficulties when using the option “never”: some respondents were unsure what was meant by never: “*if they never gave me any advice at all, can the answer be that I never got unhelpful advice?”.*
- Item 5: “Consequences” in …“understands the consequences of my health problems and illness” was inconsistently understood, depending on the situation, so they had to calculate an average when answering.
- Item 6: In “…makes me feel like I am an exaggerator” most hesitated on the formulation of the sentence and changed it automatically to “as if I am exaggerating”. Response formatting presented difficulties when using the option “never”: “*Should I answer never when I have not met this reaction”.*
- Item 7: Considering “…thinks I can work more than I do” their experience was often the opposite of the one suggested in the item, which caused hesitation.
- Item 8: In “…gives me the chance to talk about what is on my mind” “talk about” was understood as “tell” instead of “talk about”, so the committee chose a more casual word for talk, “*prata*” (Swedish), and changed the wording by adding “on” to “what I am thinking on”; this was done after contact with designer about the conceptual meaning of the expression.
- Ticking boxes (e.g., a box indicating no partner) were added to help respondents clarify why a section was skipped.
- Instructions for section 1 (spouse or partner): In section 1, most respondents were thinking with an everyday perspective when answering.
- Instructions for section 2 (family): This section was considered broad with both closest family and relatives in the same section. Often these categories of people reacted in different ways, the closest more positively and other varied much, so they had to calculate an average. The committee proposed changes in instructions for section 2 to the designer but that would have made the Swedish version differ from other language versions, so it was not changed.
- Instructions for section 3 (medical professionals), 4 (work environment), and 5 (social services): Most respondents had only met one category of personnel regarding each section, but there was no possibility to indicate which one and those who had met several categories of personnel calculated an average. In these three sections, some respondents were thinking about a longer period than was intended – often the whole illness period instead of the last year.

The overall relevancy of the content was asked for and found to be appropriate and typical of what they had experienced. The overall impression of the questionnaire was found to be clear, easy to complete and well-formulated, but section 2 (family) was difficult to answer because it could be interpreted broadly. Short sections were considered good because it gave time for reflection and opportunity to rest between questions. The same pattern throughout the sections and items facilitates a way of thinking.

In step 6, harmonization, different linguistic styles were found between the Swedish version and the other language versions but conceptually they corresponded well.

## Discussion

In this study, multiple methods were used, which is strongly advised to ensure good quality and equivalence (Acquadro et al. 2008; Maneesriwongul and Dixon 2004). Testing between-country heterogeneity is another option for finding if conceptual equivalence is retained (Acquadro et al. 2008). For this questionnaire, measurement invariance was shown in a study comparing six other translated versions of the questionnaire (Kool et al. 2013).

During the translation procedure, several difficulties were encountered with respect to concepts. Some problems were solved directly in the committee after translation but most were found during and after the cognitive interviews. Four of eight items required changes after cognitive interviews. Although translations were performed by experienced translators and reviewed by the committee, this approach still seemed lacking. The respondents pointed out difficulties during cognitive interviews. Listening to the respondents’ opinions provides a qualitative validation of instruments (Mallinson 2002) but there was no such method used in the development and validation of the original questionnaire.

Although the committee approach can result in shared misconceptions (Maneesriwongul and Dixon 2004) or pressure to form a consensus (Acquadro et al. 2008), the committee felt it necessary to include several members to obtain necessary input. The committee made their process clear with the final responsibility on the two first translators, a centralized review procedure that has been previously recommended (Acquadro et al. 2008). The use of translators with sufficient education to ensure understanding of the concepts in both languages should enhance the quality of the procedure (Sousa and Rojjanasrirat 2011).The translators and members of committee are described and qualifications were provided as an indicator of quality (Jones et al. 2001).

An instrument should include items that represent a fair sample of the construct-relevant content and cognitive interviewing is a useful method to assess the content validity (Acquadro et al. 2008; Rothman et al. 2009). In this study, the respondents considered the 3*I to have face-validity. The relevance of questionnaires’ content can influence respondents’ motivation to respond in a serious and honest manner (Knafl et al. 2007). A questionnaire like 3*I with seemingly high face-validity might be well received by potential test users.

In interviews, there is always a risk of respondents being polite to such a degree they do not share their true beliefs, being less than honest about their level of understanding (i.e., they do not ask for clarification when they are unsure what is being asked), and being discriminated against if they are less articulate than other respondents (Collins 2003). Some respondents were cautious at the start, but later they described their experiences freely and generously. Patients with a chronic pain condition were between 38 and 65 years old, which might limit the results to this group. Because more women were included in the cognitive interviews the study had a gender bias. However, the majority of patients with chronic pain conditions are women, so this sample is representative of the prospective study population. Otherwise, the study sample had good variation. Cultural adaptation will probably not preserve the psychometric properties and fully maintain the equivalency of the new Swedish version. Ideally, psychometric evaluation could be included (Acquadro et al. 2008; Maneesriwongul and Dixon 2004) but psychometric analyses are beyond the scope of this paper.

Although other studies have found that invalidation exists and is experienced (Soderberg et al. 1999; Nguyen et al. 2012) this has not been quantified. Since patients’ lived experiences are inevitably related to perceived health, invalidation might also impact compliance, results of care and treatment, quality of life, and health behaviour. With this instrument, the important invalidating components for individuals could be determined. Invalidation could be experienced before any diagnoses are determined, so using the instrument would give an idea of the situation and what health care providers are facing when meeting a patient and ultimately help health care providers develop treatments that address their patients’ experiences and needs.

## Conclusions

Following an internationally recognized methodology for translation and adaptation, we generated and tested a Swedish version of the Illness Invalidation Inventory. This study highlights the importance of using guidelines to improve the efficiency of the procedure of translation and to ensure the quality of a translated instrument and thereby its results. Psychometric analyses of validity, reliability and measurement invariance of the Swedish version will be performed in the next step. The results indicate that this questionnaire can be used in Sweden to measure experiences of invalidation and the results should provide future users of the questionnaire helpful insights into its implementation.
